# On the Use of Pessaries

**Published:** 1908-02-15

**Authors:** 


### Gynæcology.

### ON THE USE OF PESSARIES.

A few hours spent in the gynæcological department of a hospital will make it abundantly clear that no apology is needed for a discussion of the treatment of displacements of the uterus. The mere fact that numbers of women are wearing pessaries, from the use of which they receive no benefit or relief, cannot fail to convince the sceptic that the last word has not yet been written on the subject. We do not for a moment believe that we can write that word; we believe that these instruments have their legitimate uses, but that much discrimination is necessary in the selection of cases in which benefit may accrue. A pessary which is doing no good, and relieving no symptom, should be removed and some other remedy sought. At one time every uterine displacement, whatever its kind, was thus treated, and instruments of various shapes were invented by the more ingenious members of our profession, by which they hoped, no doubt, to cure their patients. The invention of so many different varieties means that the older instruments failed to cure. Most of these ingenious contrivances have been discarded now, and, in truth, where a pessary will do any good at all, the result can be achieved by a simple ring or by a Hodge.

The general disadvantages of pessaries are not always fully realised. They all, however well fitting, cause vaginal catarrh; in many cases one end of the instrument acts as a constant irritant to the cervix, and thus causes cervical catarrh. When used for prolapse, pessaries take the place of some of the natural uterine supports, in which atrophy is apt to follow. As a result of this they sometimes tend to make matters worse. The wearing of a pessary has a distinct effect on the mental state of the patient by constantly calling her attention to the generative organs. In a virgin this has a disastrous effect, and may lay the foundation of chronic neurasthenia. Those of the Zwancke type, if not removed every night, may lead to greater evils still. Deep ulceration in the lateral vaginal walls may occur, and vesico-vaginal fistula is by no means rare; these conditions may be due solely to long continued pressure, but it is also possible that some electrical action takes place where the vaginal wall touches the metal hinge of the instrument.

On the other hand, these disadvantages are not sufficiently serious to make us discard pessaries altogether. We must continue to use them in some cases, but we must adopt means to prevent their ill-effects as much as possible. By using the smallest pessary which will produce the required result, we shall limit the amount of vaginal catarrh and cervical irritation. It is quite common to meet with patients who are wearing the very largest which the vagina will hold, entailing much suffering in application and removal, and consequent neglect of this by the patient. A pessary should never stretch the vaginal walls unduly, and there should be no difficulty in passing the finger around in all directions. Frequent astringent douches will do much to wash away discharges and something towards keeping down vaginal catarrh. Removal at short intervals, and occasional abandonment for a few days, will also help in the same direction. The material is also of importance. India-rubber pessaries cause more catarrh than those made of vulcanite. Vulcanite rings, however, cannot always be used, because of the great pain and difficulty experienced in their application. It is often of value to use a pessary which is made of some malleable substance, such as pewter, so that the curves of the instrument may be varied at will to suit the needs of the patient. Pessaries of the cup and stem type, held in place by straps fastened to a waist belt, are not often used, chiefly because it is found that they often fail in their object. In some cases they are expelled from the vagina by any straining effort, unless the straps are fastened so firmly as to cause pain. This latter difficulty can be overcome by directing the waist belt to be worn outside the corsets, instead of underneath them, as so many patients attempt to do. These are only to be thought of in severe cases of uterine prolapse, where no other simple instrument will remain in the vagina.

With regard to the cases in which pessaries may be expected to relieve symptoms, in cases of backwardly displaced uteri, they may be used when the uterus is freely movable and therefore easily replaced, when there are no prolapsed tender ovaries and when the symptoms—such as backache, leucorrhœa, and perhaps menorrhagia—may be reasonably supposed to be due to chronic uterine congestion. Such cases are usually those of acquired displacements in women who have had children or abortions. If, however, the displacement is congenital, with a small uterus and a short posterior vaginal wall, as a rule no particular symptoms are caused by the displacement, and pessaries as a rule will not keep the organ in its normal position. In a virgin with this congenital backward displacement they should not be used at all. If one is used for a retroversion, let it be an absolute axiom that the uterus must be replaced before the pessary is applied. If the uterus cannot be replaced by the fingers alone, use a tenaculum to pull down the cervix first, and then replace by the fingers. The sound should be abolished as a means of replacing the uterus, except in patients under anæsthesia, in whom the vagina can be rendered aseptic as for an operation.

Whereas all cases of so-called anteflexion of the uterus, apart from pregnancy, are congenital malformations, instruments cannot be expected to relieve any symptoms, and should therefore be studiously avoided. The symptoms in such cases are due to poor development of the uterine muscle as a rule. Pessaries must be often used for cases of prolapse of varying degree of severity, often solely to make the patient comfortable. It must not be forgotten, however, that this does not cure the displacement, and it must be carefully considered whether some plastic operation may not perhaps achieve a better result.

